# Over-The-Top Technique for Revision ACL Reconstruction with Achilles Allograft and Associated Lateral Extra-articular Tenodesis

**DOI:** 10.1016/j.eats.2022.05.010

**Published:** 2022-09-21

**Authors:** Bálint Zsidai, Ian D. Engler, Oriol Pujol, Gian Andrea Lucidi, Andrew J. Curley, Stefano Zaffagnini, Volker Musahl

**Affiliations:** a Department of Orthopaedic Surgery, UPMC Freddie Fu Sports Medicine Center, University of Pittsburgh, Pittsburgh, U.S.A.; b Department of Orthopaedics, Institute of Clinical Sciences, Sahlgrenska Academy, University of Gothenburg, Gothenburg, Sweden; c Knee Surgery Unit, Orthopedic Surgery Department, Vall d'Hebron University Hospital, Universitat Autónoma de Barcelona, Barcelona, Spain; d Clinica II, Istituto Ortopedico Rizzoli, IRCCS Via Pupilli, Bologna, Italy

## Abstract

Revision anterior cruciate ligament reconstruction (ACL-R) is made challenging by the frequent presence of rotatory instability, tunnel malpositioning and widening, and limited autograft options. Lateral extra-articular tenodesis (LET), alternative tunnel routing, and the use of allograft tissue can be used to manage these challenges. This Technical Note describes revision ACL-R using the over-the-top (OTT) technique with Achilles tendon allograft with concomitant LET. The surgical approach involves routing the graft around the posterior aspect of the lateral femoral condyle, and then deep to the iliotibial band to a site just medial to Gerdy’s tubercle, with staple fixation on the lateral femur for the ACL-R and anterolateral tibia for the LET. The OTT technique with LET provides a versatile approach for the management of failed ACL-R by circumventing challenges in revision ACL-R and addressing rotatory instability, a contributing factor to prior graft failure.

## Introduction

Revision following anterior cruciate ligament reconstruction (ACL-R) presents many challenges not encountered in the primary setting. First, surgeons must assess the reasons underlying failure of the primary reconstruction. In the setting of persistent rotatory instability, associated procedures such as a lateral extra-articular tenodesis (LET) may provide added stability to the knee undergoing revision ACL-R.^1^ Second, malpositioned and widened tunnels may compromise revision tunnel placement and fixation, which may lead to staged procedures for bone grafting of the tunnels. Third, graft options may be limited due to prior autograft use and a desire to avoid additional donor site morbidity from graft harvest in the revision ACL-R patient.

The over-the-top (OTT) technique for revision ACL-R addresses each of these challenges. Initial steps involve routing the graft around the posterior aspect of the lateral femoral condyle. Subsequent graft fixation on the lateral aspect of the femoral condyle facilitates the incorporation of an LET into this technique, providing rotatory stability in the setting of a failed ACL-R. Given that no femoral tunnel is required, the OTT approach circumvents the influence of prior femoral tunnel malpositioning or widening on the revision procedure. Finally, the OTT technique permits both allograft and autograft use, providing multiple graft options in the setting of revision ACL-R.

This article presents a single-stage technique for revision ACL-R combining the OTT technique using an Achilles tendon allograft with LET augmentation, using autograft hamstring tendons, as described by Marcacci et al.^2^

## Surgical Technique

### Preoperative Planning

A thorough history and physical should be obtained prior to revision ACL-R, including an assessment of factors that led to failure of the prior graft. A standard radiographic series, along with full-length alignment films, are evaluated for prior tunnel placement, tunnel osteolysis, prior implants, joint space narrowing, and overall coronal plane and sagittal plane alignment. In addition to confirming the diagnosis of ACL insufficiency, magnetic resonance imaging (MRI) is used to assess for concomitant injuries, such as chondral damage or meniscal tears.

Prior to performing OTT ACL-R, three-dimensional computed tomography (CT) scan of the knee is recommended to provide an accurate evaluation of prior tunnel diameter and placement. The location of these prior tunnels can be categorized as 1) anatomic, 2) nonanatomic that “does not” overlap with the anatomic ACL footprint, and 3) semianatomic that “does” overlap with the anatomic footprint. Scenarios 1 and 2 are often addressed in a single-stage procedure by overreaming and then using the prior tunnel or drilling a new revision tunnel in the anatomic location, respectively. Scenario 3 is commonly managed with a two-stage procedure with bone grafting of the prior tunnels followed by a revision ACL-R several months later. However, the OTT technique provides a single-stage surgical option to scenario 3 and cases with excessive tunnel enlargement.

**Fig 1:**
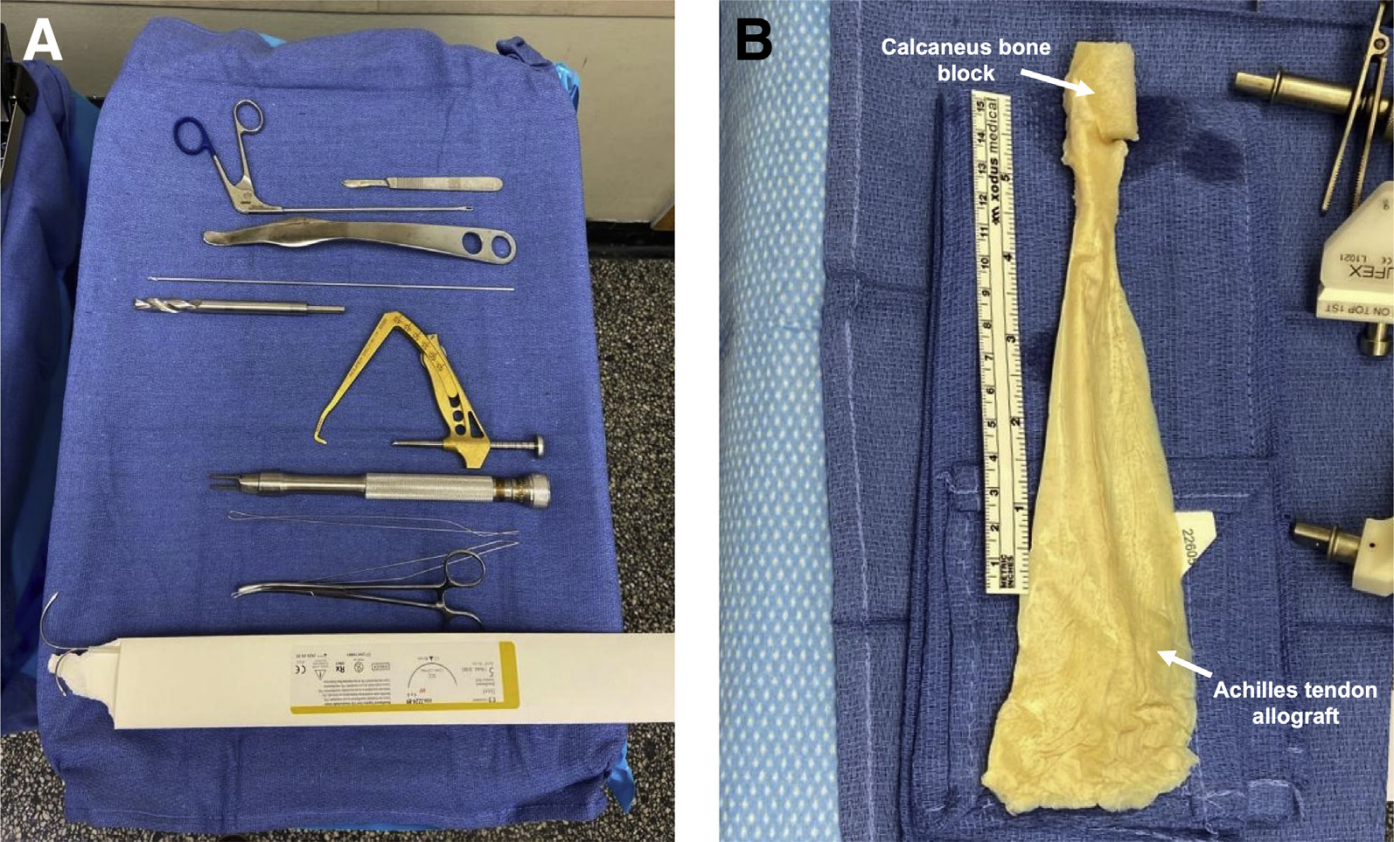
(A) Surgical equipment used in revision anterior cruciate ligament reconstruction using the over-the-top technique, From top to bottom: scalpel, loop retriever, Hohmann retractor, guidepin, straight reamer (10-11 mm), tibial tip-aimer guide, Richards staples, high-strength nonabsorbable suture, long Kelly clamp, size 5 stainless-steel monofilament sutures. (B) Achilles tendon allograft and associated calcaneus bone block (arrows).

Special equipment needed for the case includes Achilles allograft with a minimum length of 20 cm and the associated calcaneus bone block, rasp, blunt Hohmann retractor, long Kelly clamp, size 5 stainless-steel monofilament suture ×2 (needles cut off), arthroscopic loop retriever, high-strength nonabsorbable sutures, 2 large and 1 small Richards staples (Smith & Nephew, Andover, MA), ACL tibial tip-aimer guide, straight reamers, dilators, nitinol wire, PEEK interference screws (BioSure PK screw 11 mm × 25 mm, Smith & Nephew, Andover, MA), and mini C arm (Fig 1A).

### Over-the-Top ACL Reconstruction

The patient is positioned per the preferred ACL-R set-up of the surgeon. A tourniquet may be used to improve visualization.

**Fig 2:**
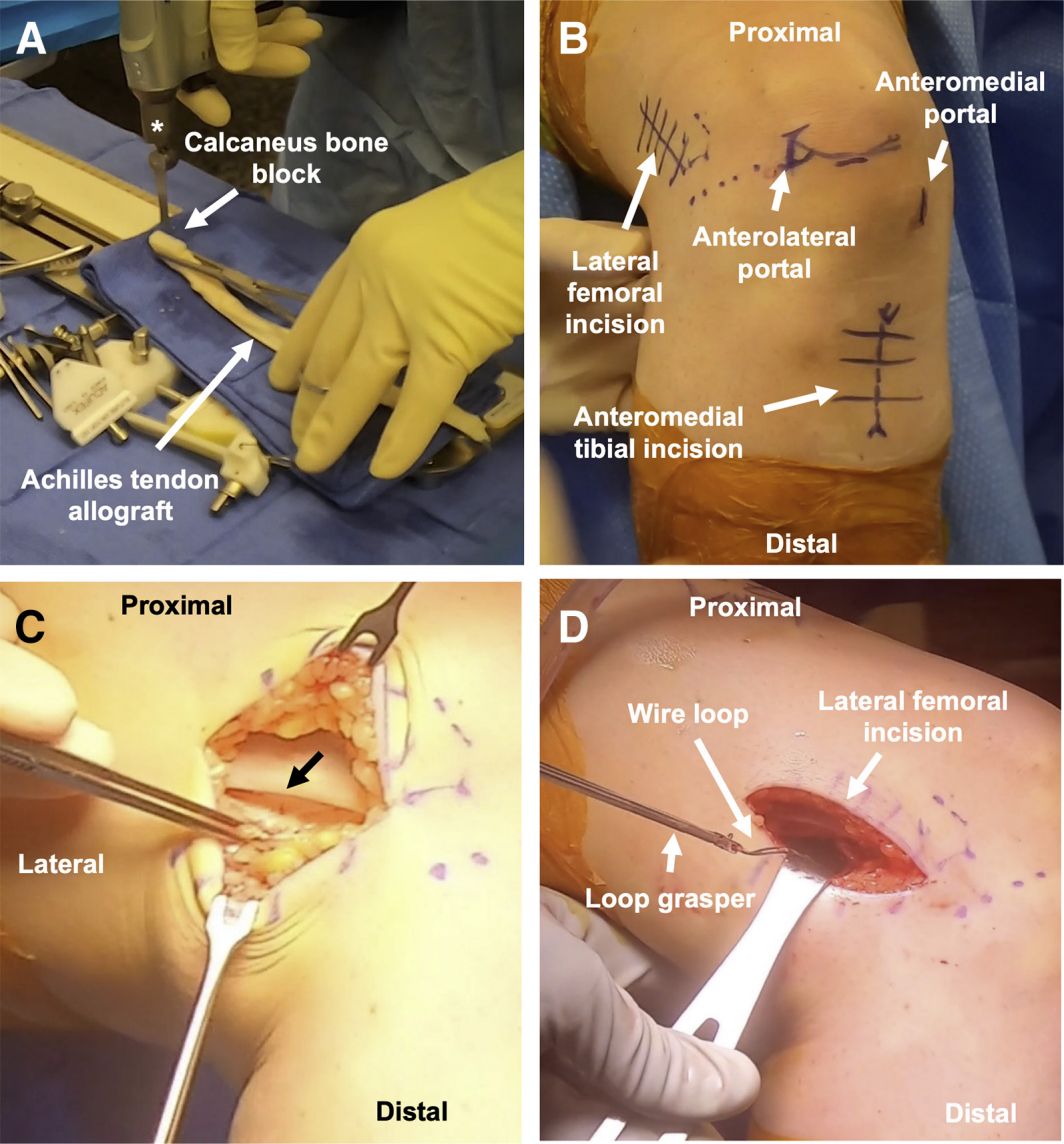
(A) The Achilles tendon allograft with associated bone block is sized to a width of 10 to 11 mm using an oscillating saw (asterisk). (B) Anterolateral view of the knee displaying the surgical incision sites (arrows) for the over-the-top technique. Lateral femoral dissection is performed along the posterior third of the iliotibial band and distal femoral metaphysis. Diagnostic arthroscopy is performed using a “high and tight” anterolateral portal. The anteromedial portal is 2 cm medial to the patellar tendon. An anteromedial tibial incision is made for tunnel placement and access to Gerdy’s tubercle. (C) Lateral view of the thigh displaying sharp incision of the posterior third of the iliotibial band (black arrow) along the length of the lateral incision. (D) Lateral view of the thigh picturing a loop grasper used to retrieve the wire loop from the over-the-top position through the lateral femoral incision.

The Achilles graft is prepared first (Fig 1B, Video 1). After thawing in warm saline for 20 minutes, the width of the calcaneus bone block is sized to 10 or 11 mm (Fig 2A). With more preoperative tibial tunnel widening, a larger bone block is used. The length of tendinous graft used is 21 cm, so the tendinous graft is cut to 23 cm in length to provide 2 cm of excess. The tendinous portion is cut beginning alongside the bone block and continuing proximally, narrowing the width of the tendinous component to about 11 mm. A no. 2 high-strength nonabsorbable suture is used to whipstitch the proximal 3 cm of tendon. The graft is wrapped in moist gauze.

Prior to arthroscopy, the lateral femur dissection is performed starting with a 4-cm longitudinal incision centered on the palpable posterior third of the IT band along the distal femoral metaphysis (Fig 2B). Following superficial dissection to the IT band, the posterior third of the IT band is sharply incised along the length of the lateral incision (Fig 2C). Blunt dissection is taken down to the posterior aspect of the lateral femoral condyle and the joint capsule.

**Fig 3:**
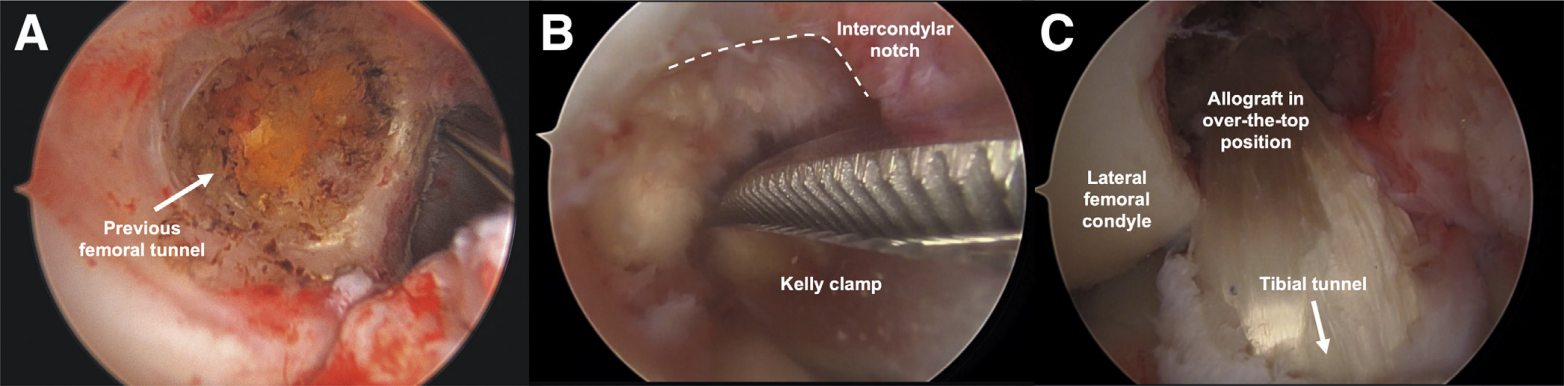
Arthroscopic images of a right knee display (A) the femoral tunnel from the failed prior ACL reconstruction (arrow), as viewed from the anteromedial portal. (B) The Kelly clamp advanced toward the over-the-top position through the intercondylar notch of the femur (dashed line). (C) The course of the revision Achilles tendon allograft through the tibial tunnel (arrow) to the over-the-top position, as viewed from the anterolateral portal.

A diagnostic arthroscopy is performed with the anterolateral portal “high and tight” to the inferior pole of the patella and edge of patellar tendon, respectively (Fig 2B). The anteromedial portal (Fig 2B) is about 2 cm medial to the patellar tendon and does not need to be as medial as with the anteromedial drilling technique. Meniscal and chondral procedures are performed as needed. The ACL graft remnant is debrided. The arthroscope can be temporarily relocated to anteromedial portal to improve visualization of the posterior aspect of the lateral femoral condyle and prior femoral tunnel (Fig 3). A size 5 steel wire suture is folded in half, with the loop grasped in the tip of a long Kelly clamp. This Kelly clamp is advanced through the anteromedial portal, into the notch (Fig 3B), and around the posterior aspect of the lateral femoral condyle, as proximally as possible. The surgeon can palpate the posterior aspect of the condyle through the lateral knee incision to assist in triangulation, ensuring that the Kelly passes through the capsule at the lateral incision (Video 1). A loop grasper pulls the wire loop out of the lateral incision (Fig 2D). A second wire loop is passed through the first and shuttled back through the anteromedial portal. A bovie and a rasp are used to expose and roughen bone along the posterior femoral condyle, along the tract of the graft.

Preparation for the tibial tunnel involves an incision over the anteromedial tibia, followed by dissection down to bone. An ACL tip-aimer guide is used to drill a guidepin from the anteromedial tibia into the anatomic tibial footprint. Straight reamers are passed over the guidepin, beginning with 8 mm and progressing to 10 mm. In the case of tibial tunnel widening, this may be dilated to 11 mm or more. A suture grasper is used to pull the intra-articular size 5 steel wire out of the anteromedial portal into the tibial tunnel.

**Fig 4:**
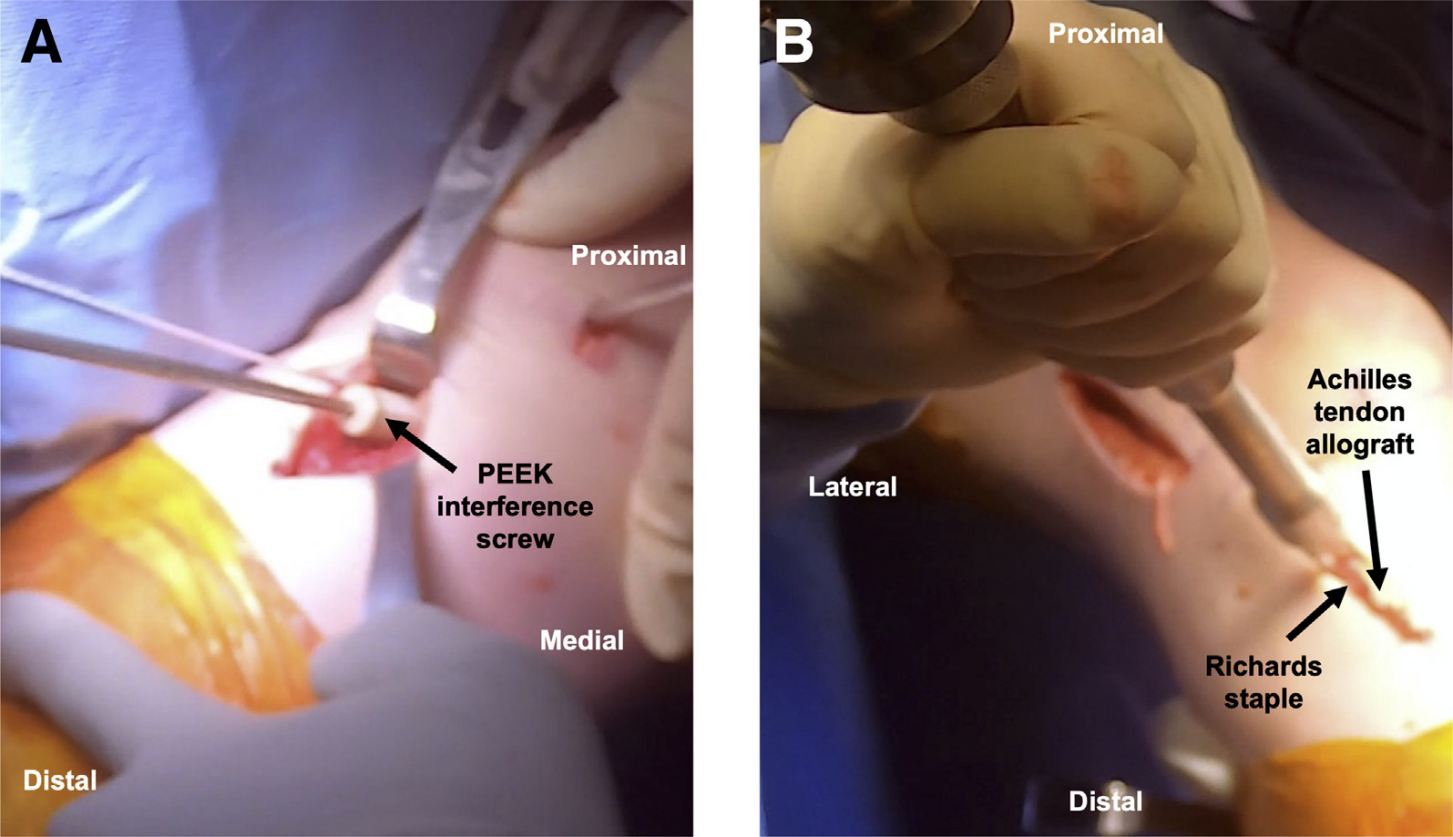
(A) Anteromedial view of the knee displaying a 25-mm length PEEK interference screw advanced over nitinol wire to secure the bone block in the tibial tunnel. (B) Lateral aspect of the knee demonstrating fixation of the remaining Achilles tendon allograft just medial to Gerdy’s tubercle with a small Richards staple.

This wire loop is used to shuttle the Achilles graft sutures over the top of the lateral femoral condyle, exiting out of the lateral incision (Video 1). The sutures are pulled such that the bone block advances into the tibial tunnel with the cancellous bone posterior and the tendinous graft anterior to anteriorize the graft into a more anatomic position. With the knee in extension (thus pulling the graft into the tunnel), the end of the bone block should be flush with the tibial cortex. The graft is held in this position, and two large Richards staples are placed over the lateral distal femur at the prepared site, posterior to the lateral epicondyle, to secure the graft. Staples should straddle the graft entirely and enter perpendicular to bone. They should be sufficiently advanced to avoid prominence but not overly advanced as to risk cutting the graft. With tension on the distal sutures, the knee is cycled for 10 repetitions of stress relaxation^3^ and placed at 20° of flexion with a posterior drawer. A 25-mm length PEEK interference screw with the same diameter as the tunnel is advanced over a nitinol wire, which is placed posterior to the bone block (Fig 4A). Visualization of the graft in knee extension should ensure no graft impingement anteriorly.

### Lateral Extra-articular Tenodesis

**Fig 5:**
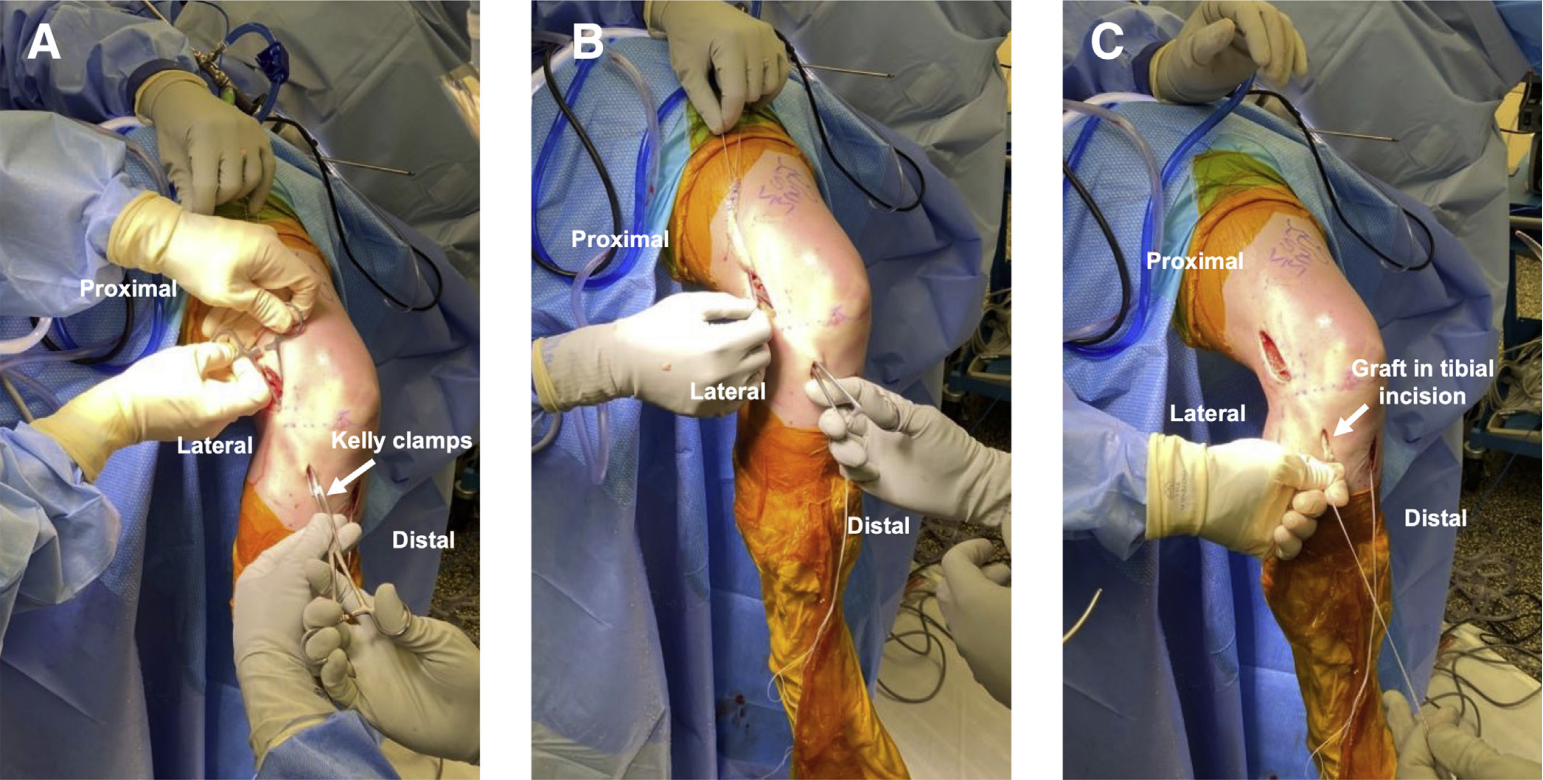
Passing the lateral extra-articular tenodesis graft in a right knee. (A) A Kelly clamp (arrow) tunneled under the iliotibial band from proximal to distal incisions is used to shuttle a second (distal) Kelly clamp proximally. (B) The Achilles allograft sutures are grasped in the Kelly clamp and (C) pulled distally into the tibial incision.

**Fig 6:**
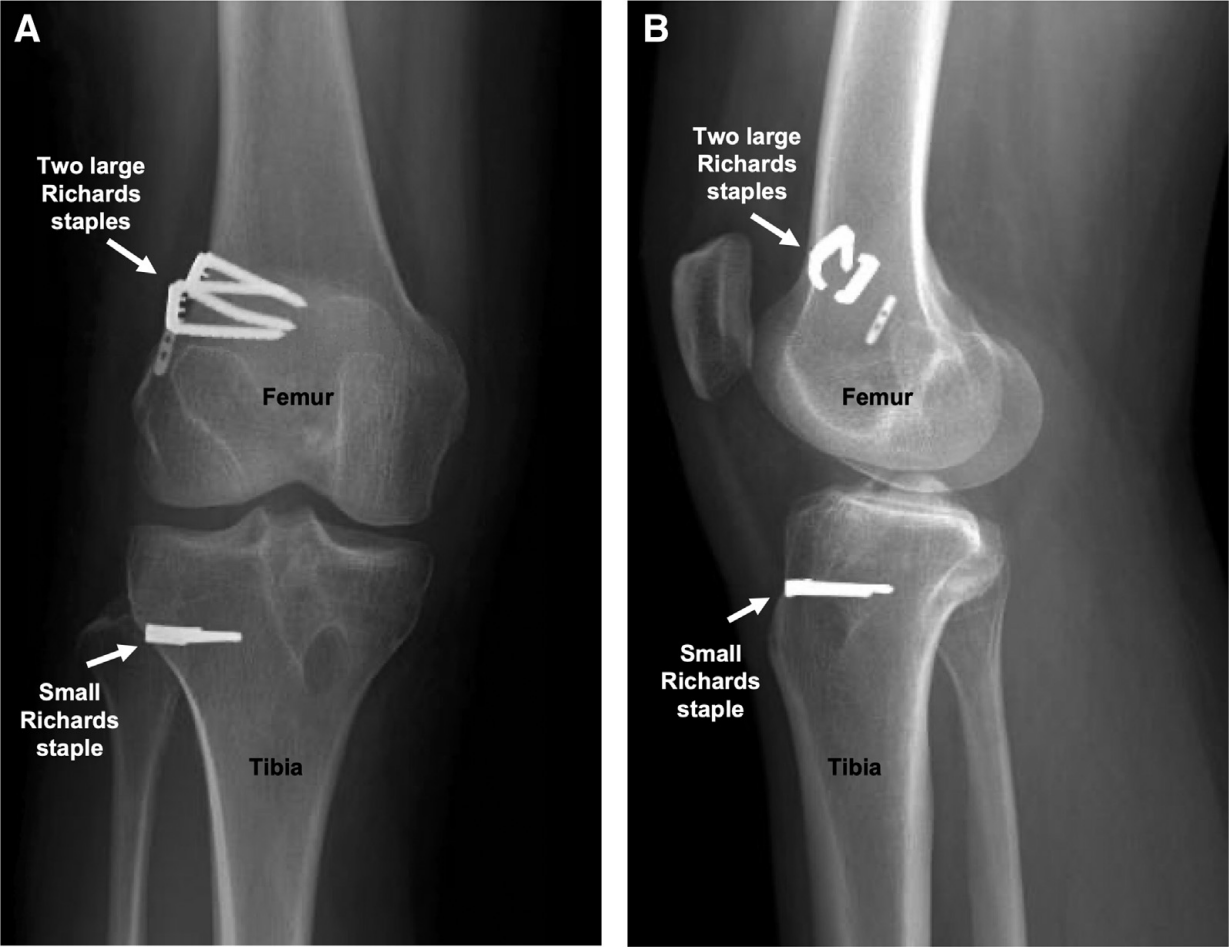
Femoral and tibial fixation of the Achilles tendon allograft using Richards staples (arrows) is verified on anteroposterior (A) and lateral (B) right knee radiographs.

A longitudinal 2-cm anterolateral tibial incision is performed medial to Gerdy’s tubercle, with dissection down to bone (Video 1). Kelly clamp tunnels under the IT band from the proximal to distal incisions, which is then interlocked with a second Kelly clamp that is shuttled back proximally. The graft sutures are passed distally out of the incision and tensioned to pull the remaining Achilles allograft into the tibial incision (Fig 5). A spinal needle is inserted percutaneously at the joint line, providing a reference to ensure that the staple is not placed too proximally. The knee is placed in 60° of flexion in neutral rotation, tension is placed on the graft, and a small staple is placed over the graft just medial to Gerdy’s tubercle (Fig 4B). A mini C arm is used to confirm staple position (Fig 6). The remaining graft stump is cut. The wounds are irrigated and closed in standard fashion. Postoperative protocol and rehabilitation may proceed, according to surgeon preference for allograft revision ACL-R.

## Discussion

**Fig 7:**
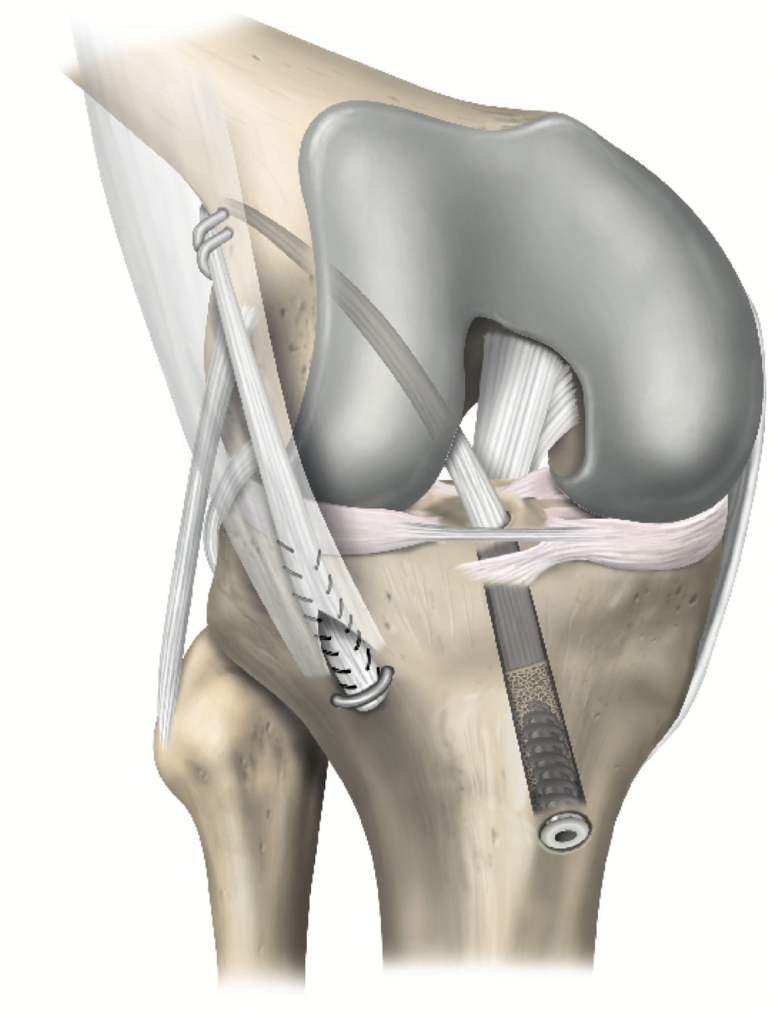
Schematic illustration of the over-the-top anterior cruciate ligament reconstruction technique performed using an Achilles tendon allograft with bone block and lateral extra-articular tenodesis.

Revision ACL-R may be challenging in the setting of extensive femoral tunnel osteolysis or overlap between prior and planned tunnels. Two-stage revision with bone grafting addresses these issues but exposes the patient to increased risk from a second procedure, increased recovery time, and increased cost. The OTT ACL-R technique is a single-stage technique for revision ACL-R that bypasses each of these issues by elimination of the need to drill femoral tunnels. Furthermore, the excess graft can be incorporated into a LET for additional rotatory control, which is often beneficial in the revision setting (Fig 7). Additionally, the over-the-top technique offers a value-based method for revision ACL-R, since only 3 staples are required for graft and LET fixation at the femur and tibia, respectively.

Revision ACL-R is accompanied by a higher failure rate and decreased activity levels compared with primary ACL-R, and these worse outcomes are compounded in the multiple revision ACL-R.^4^ While two-stage revision ACL-R with bone grafting demonstrates adequate outcomes, an additional procedure requires an interval of 3-4 months for graft healing to occur. Beyond a delayed recovery and risks inherent to a second surgery, the patient is vulnerable to developing additional intra-articular injuries due to residual knee instability during this time period.^5^ Application of the OTT technique in a revision setting provides a versatile alternative to traditional approaches and has been shown to have a 52% rate of return to preinjury levels of sport, an 8.4% failure rate,^6^ and functional outcomes equivalent to those of anatomic single- and double-bundle revision ACL-R.^7^

Despite the existing controversy regarding the use of extra-articular augmentation concurrent to ACL-R, revision patients with a high-grade pivot shift and a predisposition to graft failure may benefit from the addition of LET.^8^ A prospective study investigating the use of the OTT approach in combination with LET demonstrated favorable long-term clinical outcomes in 86% of patients.^9^ In the setting of multiple revision ACL-Rs, autogenous graft choices may be exhausted or imply further donor-site morbidity for the patient. Although a topic of debate, some studies show that the risk of graft failure following revision ACL-R using allograft is comparable to that of autograft.^10^^,^^11^ Consequently, the use of allograft tissue in combination with the OTT technique is a viable option for revision ACL-R patients, especially in lower-demand patients. An Achilles allograft with a bone block allows rotation of the graft within the tibial tunnel and alignment of the tendinous portion of the graft with the anatomic ACL footprint.^12^ Alternatively, doubled autograft hamstring tendon autograft may be used and often provides enough length to span from the anterior tibia around the lateral femoral condyle to the LET attachment on the tibia.^9^

While the many benefits of OTT revision ACL-R (Table 1) outweigh the potential disadvantages of this technique, opponents have raised concerns regarding the nonisometric function and nonanatomic position of the graft.^13^^,^^14^ We believe that the OTT graft is, in fact, quite anatomic by placing the graft so posterior on the lateral femoral condyle.^15^ Furthermore, isometry should not be a goal of ACL-R, given that the ACL is not an isometric structure.^16^

**Table 1:** Advantages and Disadvantages of the Over-the-Top Technique of Revision ACL Reconstruction With Achilles Allograft and Associated Lateral Extra-Articular Tenodesis

Advantages	Disadvantages/Risks
• Eliminates concerns with femoral tunnel malpositioning, widening, and hardware removal	• Extra incision
• Avoids the risks and prolonged recovery associated with two-stage revision ACL-R	• Allograft use, with potentially increased risk of graft failure
• Restores rotatory stability	• Possible symptomatic hardware
• Unaffected by previous graft harvest	
• Cost-effective graft fixation	

In conclusion, this Technical Note describes the surgical technique for OTT revision ACL-R with Achilles tendon allograft with an associated LET. The OTT approach provides a versatile method for the surgical treatment of recurrent ACL tears (Table 2). Importantly, the surgical technique described in this article is able to eliminate several challenges faced by the surgeon during revision ACL-R, including femoral tunnel malpositioning and widening, a lack of remaining options for ipsilateral graft harvest, and residual rotatory instability.

**Table 2:** Pearls and Pitfalls of the Over-the-Top Technique of Revision ACL Reconstruction With Achilles Allograft and Associated Lateral Extra-Articular Tenodesis

Pearls	Pitfalls
• Indicated in patients with partially or nearly overlapping prior and planned femoral tunnels and/or extensive femoral tunnel widening	• Lateral knee dissection should not be in biceps femoris plane
• Prior femoral hardware may be removed, and prior tunnels may be bone grafted per surgeon preference	• Do not compromise tibial tunnel placement by using a malpositioned prior tunnel
• Achilles allograft bone block is sized based on tibial tunnel width; tendinous portion is cut to 23 cm length and tubularized	• Avoid skiving staples into bone or piercing the graft with the staple
• Graft placement site at the posterior lateral femoral condyle is rasped to stimulate healing	• Leaving staples proud may not secure the graft or irritate the IT band
• Staples for graft fixation should be perpendicular to the bone without piercing the graft, extra-articular, and appropriately seated, as confirmed on C arm	• Graft-tunnel mismatch may occur if the graft is fixed at the lateral femoral condyle with the bone block extruding from the tibial tunnel
• Order of fixation: staples at lateral femoral condyle, interference screw at tibial tunnel (20° of flexion and posterior drawer), staple just medial to Gerdy’s tubercle (60° of flexion in neutral rotation)	• Do not place tibial staple too close to the articular surface

## Supplementary Data

ICMJE author disclosure forms

Video 1 This video demonstrates the over-the-top (OTT) technique for revision anterior cruciate ligament (ACL) reconstruction with an associated lateral extra-articular tenodesis (LET). The procedure begins with preparation of the allograft. The tendinous portion of the graft is cut to length and a diameter of 10 or 11 mm. The end of the graft is bulletized and a 10- or 11-mm diameter bone block is prepared. An incision is made at the distal aspect of the lateral thigh, taken down to the IT band, and flaps are developed. An incision is made longitudinally, 1 centimeter anterior to the posterior aspect of the IT band. Cautery is used to maintain hemostasis. Dissection is taken down to the posterior aspect of the lateral femoral condyle. Diagnostic arthroscopy may then be performed. The remnant ACL graft is debrided. A Kelly clamp is advanced through the anteromedial portal and around the posterior aspect of the lateral femoral condyle in the OTT position. The Kelly clamp holds the end of a looped wire, which is then grasped out of the lateral thigh incision. As displayed, the prior ACL tunnel with the associated graft is malpositioned. The tibial tunnel is drilled in the anatomic position. A wire shuttles a suture, and this is withdrawn down the tibia. A suture is then used to shuttle the graft into the OTT position, and the graft is secured over the lateral aspect of the distal femur, just posterior to the lateral epicondyle, using two Richards staples (Smith & Nephew, Andover, MA). Further fixation is achieved with a second staple placed just distal to the first. An incision is then made just medial to Gerdy’s tubercle, and dissection is then taken down to the bone. Kelly clamps are used to define the tract for graph passage deep to the IT band from the proximal to distal incision. Kelly clamps are used to shuttle the suture and graft into the distal incision. Tension is placed on the tibial ACL sutures, and the graft is cycled with the knee at 20° of flexion and posterior drawer, as well as firm tension on the tibial ACL sutures. An interference screw (BioSure PK screw 11 mm × 25 mm, Smith & Nephew, Andover, MA) is placed for tibial graft fixation. The tibial aspect of the LET is fixed using a small Richards staple, which must be perpendicular to the bone, and extra-articular. The IT band and skin are closed in a standard layered fashion.
